# Thickness-dependent in-plane anisotropy of GaTe phonons

**DOI:** 10.1038/s41598-021-00673-0

**Published:** 2021-10-27

**Authors:** Nguyen The Hoang, Je-Ho Lee, Thi Hoa Vu, Sunglae Cho, Maeng-Je Seong

**Affiliations:** 1 Department of Physics, Chung-Ang University, Seoul, 06974 Republic of Korea; 2 Department of Physics and Energy Harvest Storage Research Center, University of Ulsan, Ulsan, 44610 Republic of Korea; 3 Center for Berry Curvature-Based New Phenomena, Chung-Ang University, Seoul, 06974 Republic of Korea

**Keywords:** Materials science, Physics

## Abstract

Gallium Telluride (GaTe), a layered material with monoclinic crystal structure, has recently attracted a lot of attention due to its unique physical properties and potential applications for angle-resolved photonics and electronics, where optical anisotropies are important. Despite a few reports on the in-plane anisotropies of GaTe, a comprehensive understanding of them remained unsatisfactory to date. In this work, we investigated thickness-dependent in-plane anisotropies of the 13 Raman-active modes and one Raman-inactive mode of GaTe by using angle-resolved polarized Raman spectroscopy, under both parallel and perpendicular polarization configurations in the spectral range from 20 to 300 cm^−1^. Raman modes of GaTe revealed distinctly different thickness-dependent anisotropies in parallel polarization configuration while nearly unchanged for the perpendicular configuration. Especially, three A_g_ modes at 40.2 (${\text{A}}_{\text{g}}^{1}$), 152.5 (${\text{A}}_{\text{g}}^{7}$), and 283.8 (${\text{A}}_{\text{g}}^{12}$) cm^−1^ exhibited an evident variation in anisotropic behavior as decreasing thickness down to 9 nm. The observed anisotropies were thoroughly explained by adopting the calculated interference effect and the semiclassical complex Raman tensor analysis.

## Introduction

Two-dimensional (2D) materials such as graphene and transition metal dichalcogenides (TMDCs) attracted massive attention. These materials frequently displayed diverse optical and electronic properties with a high in-plane isotropy^1–3^. However, a new category of 2D layered materials exhibiting in-plane anisotropic physical properties, such as black phosphorus (BP), WTe_2_, TlSe, ReS_2_, ReSe_2,_ and SnSe^4–11^, has recently emerged. Generally, the in-plane anisotropy in electrical, optical, thermal, and phonon properties is associated with the low in-plane symmetry of crystal structure. By manipulating the in-plane anisotropy, we can separately tune and optimize these properties along desired crystallographic orientations. Thus, the in-plane anisotropy provides a tremendous opportunity for designing various polarization-resolved devices, including anisotropic field-effect transistors (FETs), polarization-sensitive photodetectors, integrated polarization controllers, surface-enhanced Raman scattering devices, and linearly-polarized ultrafast lasers^5,12–18^. Moreover, in-plane anisotropic properties are normally originated from the different energy band structure along different in-plane directions. Therefore, the comprehensive studies on anisotropic properties of low-symmetry 2D materials could offer more insights into their energy band structures and physical properties.

Gallium Telluride (GaTe), a member of the low-symmetry material family with monoclinic structure, has gained lots of attention in recent years. Bulk GaTe has a direct bandgap of 1.65 eV at room temperature^19^, and it has extremely high photoresponsivity of 10^4^ A/W^20^. Besides, its monoclinic structure leads to anisotropic optical^21–23^ and electrical properties^24^. Among many experimental methods used to study its anisotropic properties, the angle-resolved polarized Raman spectroscopy (ARPRS) was quite useful to investigate the in-plane polarization anisotropy of its phonon modes^22,25^. Similar to BP and SnS^26,27^. Each Raman mode of GaTe crystal was reported to have peculiar polarization anisotropies that depend on its thickness and the excitation laser energy^22^. Nonetheless, systematic study on the layer-thickness-dependent in-plane anisotropy of GaTe phonon modes is yet to be done.

In this work, we have investigated the in-plane anisotropies of the 13 Raman-active modes and one Raman-inactive mode of GaTe flakes with different thicknesses by using ARPRS in the spectral range from 20 to 300 cm^−1^. Phonon modes of GaTe revealed distinctly different thickness-dependent anisotropies. The observed anisotropies can be thoroughly explained by adopting the calculated interference effect and the semiclassical complex Raman tensor analysis.

## Results and discussion

**Figure 1:**
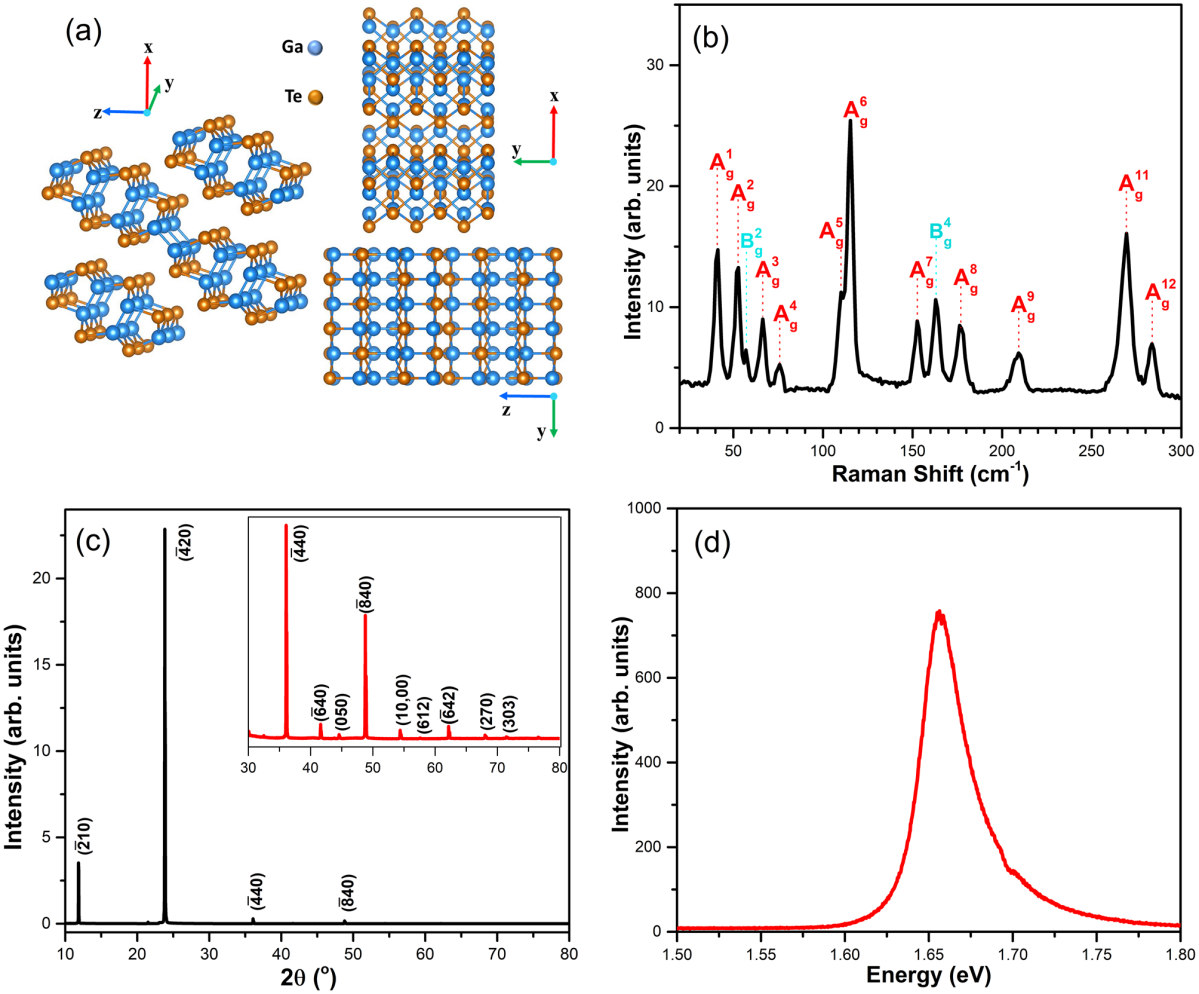
(**a**) Crystal structure of GaTe, shown in perspective view, side view, and top view of three stacked layers. The drawing was created by using VESTA version 3 (https://jp-minerals.org/vesta/en/download.html). (**b**) Typical Raman spectrum of bulk-GaTe measured by using a 532 nm laser line at room temperature. (**c**) XRD patterns of GaTe. (**d**) PL spectrum of bulk-GaTe at room temperature.

The crystal structure of bulk-GaTe is shown in Fig. 1a. It has a monoclinic structure whose space group is C2/m. In this work, we will use the conventional crystallographic axes of GaTe, where the x-axis is the direction of the stacked layers, also known as [$\overline{2 }01$] direction^25,28^. On the layer plane, the y-axis is the direction of a series of Ga-Ga binding chains. Thus, the y-axis and z-axis correspond to [010] and [102] directions, respectively. Typical Raman spectrum of our GaTe sample was shown in Fig. 1b, where eleven A_g_ modes and two B_g_ modes were observed in the spectral range from 20 to 300 cm^−1^. It is noteworthy that our Raman spectrum did not show peaks around 132 cm^−1^ and 146 cm^−1^, which were known to exist in oxygen-contaminated GaTe samples^28,29^.

The XRD spectrum of bulk GaTe has two strong peaks corresponding to ($\overline{2}10$) and ($\overline{4}20$) planes as shown in Fig. 1c, confirming that our GaTe sample is a monoclinic single-crystal^30–32^. Typical room temperature photoluminescence (PL) spectrum was shown in Fig. 1d, where a strong peak at 1.66 eV, in good agreement with the previously reported direct bandgap of GaTe, was observed^28,29,33^. All the results shown in Fig. 1 confirmed that our samples were high-quality monoclinic single-crystal GaTe.

**Figure 2:**
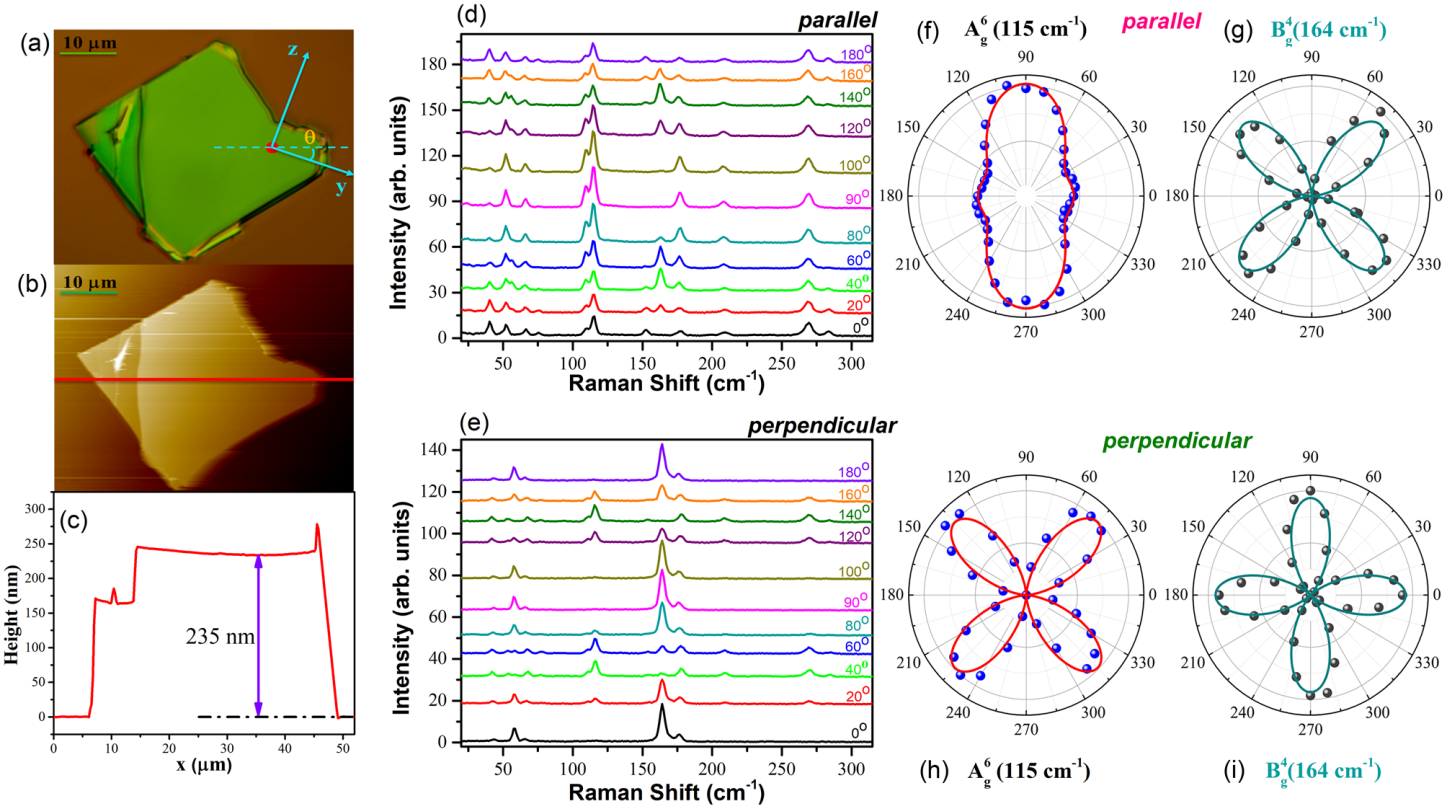
(**a**) Optical microscope image of the measured GaTe flake, where the crystal orientation and the angle θ between the polarization of the incident light and the y-axis were indicated. (**b,c**) AFM image and height profile of the GaTe flake. Polarization-resolved Raman spectra of a thick flake under (**d**) parallel and (**e**) perpendicular polarization configurations. Raman intensity polar plots of ${A}_{g}^{7}$ (115 cm^−1^) and ${B}_{g}^{4}$ (164 cm^−1^) modes under (**f,g**) parallel and (**h,i**) perpendicular polarization configurations, where solid lines represent fitted curves.

The optical microscope (OM) image and the AFM image of the mechanically exfoliated thick GaTe flake were shown in Fig. 2a,b, respectively. The thickness of the measured flake was ~ 235 nm as shown in Fig. 2c. Raman-active modes in GaTe consisted of 12 A_g_ and 6 B_g_ modes, and all the phonon modes, including the Raman-inactive modes, are shown in Table S1^22,23^. 13 Raman-active modes were observed in this work and their Raman spectra with differing angles of polarization direction in both parallel and perpendicular polarization configurations were shown in Fig. 2d,e, respectively. In the parallel polarization configuration, the Raman intensities of the A_g_ modes exhibited maxima for the angle θ between the polarization direction and the crystal y-axis of 0°_,_ 90°, 180° and 270°, whereas those of the B_g_ modes vanished for θ = 0°_,_ 90°, 180° and 270°. Typical polarization anisotropies of the A_g_ and the B_g_ modes in the parallel polarization configuration were shown in Fig. 2f,g, respectively. In the perpendicular polarization configuration, the intensities of the A_g_ modes became very weak or even undetectable, while the intensities of the B_g_ modes became relatively strong. Typical polarization anisotropies of the A_g_ and the B_g_ modes in the perpendicular polarization configuration were shown in Fig. 2h,i, respectively, where the A_g_ modes exhibited maxima at θ = 45°_,_ 135°, 225° and 315° and minima at θ = 0°_,_ 90°, 180° and 270° while the B_g_ modes exhibited maxima at θ = 0°_,_ 90°, 180° and 270° and minima at θ = 45°_,_ 135°, 225° and 315°. The observed polarization anisotropies can be explained by using Raman tensor analysis.

Theoretically, the intensity of a Raman-active mode can be calculated as^34^

1 $${\text{I}} \propto \left| {{\text{e}}_{{\text{i}}} {\text{Re}}_{{\text{s}}} } \right|^{{2}} ,$$

where e_i_ and e_s_ are the unit vectors of the polarization of the incident and the scattered lights, respectively, and R represents the Raman tensor for the Raman-active phonon mode. For the incident light with its polarization direction making angle θ with respect to the crystal y-axis, e_i_ = (0 cosθ sinθ) and e_s_ = (0 cosθ sinθ) or e_s_ = (0 − sinθ cosθ) for parallel or perpendicular polarization configurations, respectively. Similar to BP^9^, the Raman tensor elements of GaTe are complex numbers, and the Raman tensors for the A_g_ and the B_g_ modes of GaTe can be expressed as

$$\text{R}\left(\text{Ag}\right)=\left(\begin{array}{ccc}\left|\text{a}\right|{e}^{i{\uppsi }_{\text{a}} }& \left|\text{d}\right|{e}^{i{\uppsi }_{\text{d}} }& 0\\ \left|\text{d}\right|{e}^{i{\uppsi }_{\text{d}} }& \left|\text{b}\right|{e}^{i{\uppsi }_{\text{b}} }& 0\\ 0& 0& \left|\text{c}\right|{e}^{i{\uppsi }_{\text{c}} }\end{array}\right) \text{and R}\left(\text{Bg}\right)=\left(\begin{array}{ccc}0& 0& \left|\text{e}\right|{e}^{i{\uppsi }_{\text{e}} }\\ 0& 0& \left|\text{f}\right|{e}^{i{\uppsi }_{\text{f}} }\\ \left|\text{e}\right|{e}^{i{\uppsi }_{\text{e}} }& \left|\text{f}\right|{e}^{i{\uppsi }_{\text{f}} }& 0\end{array}\right),$$

where ψ_a_, ψ_b_, ψ_c_, ψ_d_, ψ_e_, ψ_f_ are the phases of Raman tensor elements, and |a|, |b|, |c|, |d|, |e|, |f| are amplitudes of Raman tensor elements. The calculated Raman intensities for the A_g_ and the B_g_ modes in both polarization configurations are shown below.

2 $${\text{I}}\left( {A_{g}^{//} } \right)\sim \left| {\text{b}} \right|^{{2}} {\text{cos}}^{{4}} \uptheta + \, \left| {\text{c}} \right|^{{2}} {\text{sin}}^{{4}} \uptheta + { 2}\left| {\text{b}} \right|\left| {\text{c}} \right|{\text{cos}}^{{2}} \uptheta \;{\text{sin}}^{{2}} \uptheta \;{\text{cos}}\uppsi_{{{\text{bc}}}} ,$$

3 $${\text{I}}\left( {B_{g}^{//} } \right)\sim ({4}\left| {\text{f}} \right|^{{2}} {\text{cos}}^{{2}} \uptheta \;{\text{sin}}^{{2}} \uptheta ),$$

4 $${\text{I}}\left( {A_{g}^{ \bot } } \right)\sim \left| {\text{c}} \right|^{{2}} {\text{cos}}^{{2}} \uptheta \;{\text{sin}}^{{2}} \uptheta + \, |{\text{b}}|^{{2}} {\text{sin}}^{{2}} \uptheta \;{\text{cos}}^{{2}} \uptheta - {2}\left| {\text{b}} \right|\left| {\text{c}} \right|{\text{cos}}^{{2}} \uptheta \;{\text{sin}}^{{2}} \uptheta \;{\text{cos}}\uppsi_{{{\text{bc}}}} \sim {\text{sin}}^{{2}} {2}\uptheta ,$$

5 $${\text{I}}\left( {B_{g}^{ \bot } } \right)\sim \left( {\left| {\text{f}} \right|^{{2}} {\text{cos}}^{{2}} {2}\uptheta } \right),$$

where // and ⊥ represent parallel and perpendicular polarizations, respectively, and ψ_bc_ = ψ_b_ − ψ_c_ is the phase difference.

Similar to other well-known anisotropic materials^6,9,35^, the observed polarization anisotropy of the Raman-active phonon modes of GaTe shown in Fig. 2 can be perfectly explained by using the semi-classical theory with complex Raman tensors.

**Figure 3:**
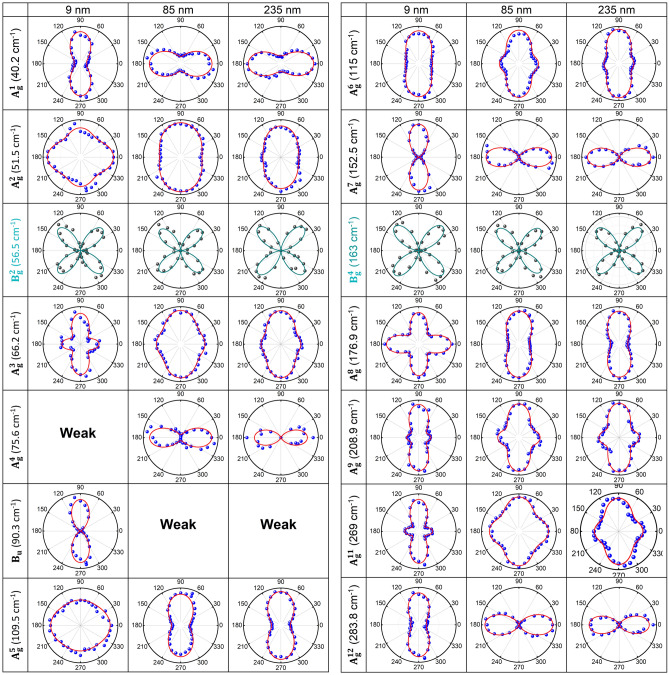
The Raman intensity polar plots and fitted curves of the observed 12 Raman-active modes from three different-thickness GaTe flakes in parallel polarization configuration. The blue (A_g_) and the gray (B_g_) dots are experimental values and the solid red (A_g_) and dark cyan (B_g_) lines are fitted curves.

To further understand the polarization anisotropy of the Raman-active modes in GaTe with different flake thickness, we carried out ARPRS in both polarization configurations on three flakes with thicknesses of 9, 85, 235 nm. The OM images, AFM images, and AFM height profiles of the 9 nm and 85 nm flakes were shown in Fig. S1, and the ARPRS spectra were shown in Fig. S2. The Raman intensity polar plots and fitted curves of the observed 13 Raman-active modes from the three different-thickness GaTe flakes in parallel and perpendicular polarization configurations were shown in Fig. 3 and Fig. S3, respectively. Especially, in parallel polarization configuration, the obtained intensity polar plot of B_u_ Raman-inactive mode at 90.3 cm^−1^ is supposed to be a variation of the symmetrical structure for a very thin GaTe-flake at 9 nm^36,37^. For 85 nm and 235 nm flakes, the observed polarization anisotropies of all the Raman-active modes are more or less the same as shown in Fig. 3. However, in parallel polarization configuration, 9 nm flake exhibited distinctly different polarization anisotropy for the A_g_ modes from the other thicker flakes, whereas its polarization anisotropy for the B_g_ modes is almost the same as that observed from the other thicker flakes. In contrast, in perpendicular polarization configuration, the observed polarization anisotropies for both the A_g_ and the B_g_ modes were almost the same for the three flakes regardless of their thicknesses, which is consistent with Raman tensor analysis as shown in the results below Fig. S3. The observed difference in polarization anisotropy from different thickness flakes can be fully explained by invoking the optical absorption, birefringence effect, and phase differences in Raman tensor elements for GaTe^9,26,38^, and the detailed analysis was described in the Supporting Information. These results demonstrated that the detailed polarization anisotropies of the Raman-active modes depend on not only its phonon mode symmetry but also its thickness.

**Figure 4:**
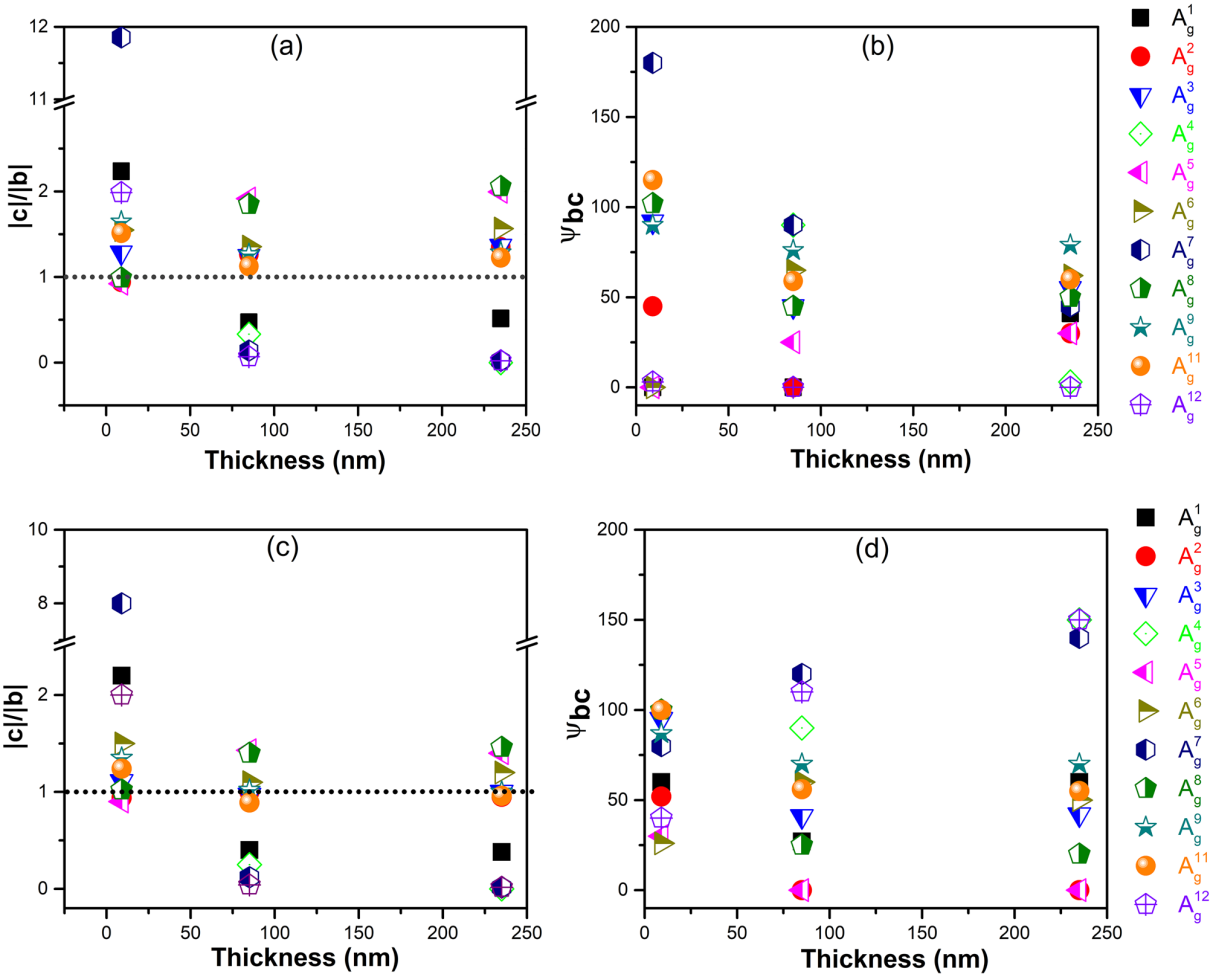
Thickness dependence of the amplitude ratio of Raman tensor elements $\frac{|c|}{|b|}$, (**a,c**), and phase difference ψ_bc_ =|ψ_b_ − ψ_c_| (**b,d**), for all A_g_ modes measured in parallel polarization configuration. The horizontal dotted lines in (**a,c**) indicates $\frac{|c| }{|b|}$ = 1. (**a,b**) Are the results before the elimination of the enhancement factor, and (**c,d**) are the results after the elimination of the enhancement factor.

To investigate the in-plane anisotropic Raman behavior further, we analyze the amplitude ratio $\frac{|\text{c}|}{|\text{b}|}$ of Raman tensor elements and ψ_bc_ phase difference for all A_g_ modes, by applying Eq. (2) for fitting experimental Raman polar plots as shown in Fig. 3. According to semiclassical Raman theory^9^, |b|${e}^{i{\uppsi }_{\text{b}}}$ and |c|${\text{e}}^{i{\uppsi }_{\text{c}}}$ characterize the Raman intensities of the phonon modes along y-axis and z-axis, respectively, in parallel polarization configuration. From Eq. (2), $\frac{|\text{c}|}{|\text{b}|}$ >1 or $\frac{|\text{c}|}{|\text{b}|}$ <1 determines whether the main polarization of corresponding phonon mode aligns along z-axis or y-axis, while $\frac{|\text{c}|}{|\text{b}|}$ = 1 indicates either isotropic Raman mode or fourfold symmetry along both y-axis and z-axis, and ψ_bc_ is related to the strength the secondary maximum in the polar plot of A_g_ Raman-active modes with twofold symmetry. In Fig. 4a,b, the ratio $\frac{|\text{c}|}{|\text{b}|}$ in three A_g_ Raman-active modes, ${\text{A}}_{\text{g}}^{1}$, ${\text{A}}_{\text{g}}^{7}$, and ${\text{A}}_{\text{g}}^{12}$, exhibited distinct thickness-dependent variation, similar to that reported in SnS^27^, while the amplitude ratio $\frac{|\text{c}|}{|\text{b}|}$ of the other A_g_ Raman-active modes did not show any definite thickness dependence. In this context, it is worthwhile to note that Zhao et al.^39^ reported the crystal structure of GaTe changes from monoclinic to hexagonal lattice as its layer thickness decreases to a few layers, which can be readily recognized by the polarization anisotropy differences in A_g_ modes between 9 nm flake and thick flakes (85 nm and 235 nm) as shown in Fig. 3. For the thick flakes (85 and 235 nm), ${\text{A}}_{\text{g}}^{1}$, ${\text{A}}_{\text{g}}^{7}$, and ${\text{A}}_{\text{g}}^{12}$ modes, whose $\frac{|\text{c}|}{|\text{b}|}$ values are rather larger than the other A_g_ modes, exhibited twofold symmetry with intensity maxima along y-axis whereas ${\text{A}}_{\text{g}}^{2}$, ${\text{A}}_{\text{g}}^{3}$, ${\text{A}}_{\text{g}}^{5}$, ${\text{A}}_{\text{g}}^{6}$, ${\text{A}}_{\text{g}}^{8}, {\text{A}}_{\text{g}}^{9}$, and ${\text{A}}_{\text{g}}^{11}$ showed twofold symmetry with intensity maxima along z-axis or fourfold symmetry. Thus, the emergence of the Raman-forbidden B_u_ mode in bulk GaTe at 90.3 cm^−1^ and the substantial change in Raman anisotropy for ${\text{A}}_{\text{g}}^{1}$, ${\text{A}}_{\text{g}}^{7}$, and ${\text{A}}_{\text{g}}^{12}$ modes, as the layer thickness decreases from 85 to 9 nm, can be ascribed to the crystal symmetry change from monoclinic to hexagonal.

As revealed in black phosphorus^26^, the interference effect contributed significantly to the thickness dependence of Raman anisotropy, and it may also play a role in GaTe. To appraise the importance of this effect, we calculated the enhancement factor of A_g_ modes along the y- and z-axis, and the details are presented in Supporting Information. As shown in Fig. S5 in Supporting Information, the ratio of the enhancement factors between the y-axis and z-axis depends significantly on GaTe thickness. Figure 4c,d showed the $\frac{|\text{c}|}{|\text{b}|}$ and ψ_bc_ after the elimination of interference effect in Raman intensity, representing intrinsic anisotropy of GaTe originating from anisotropic electron–phonon and electron-photon interactions. Overall anisotropy was reduced after correcting the interference effect but the thickness-dependent anisotropy variation for the three A_g_ modes, ${\text{A}}_{\text{g}}^{1}$, ${\text{A}}_{\text{g}}^{7}$, and ${\text{A}}_{\text{g}}^{12}$, still distinctly remained as compared to that for the rest of the A_g_ modes.

## Conclusions

In summary, we systematically investigated the in-plane anisotropy of GaTe phonon modes under parallel and perpendicular polarization configurations. 13 out of the total 16 Raman-active modes, 11 A_g_ modes and 2 B_g_ modes, were observed. Besides, one Raman-inactive B_u_ mode was also observed only in a very thin flake. Three A_g_ modes, ${\text{A}}_{\text{g}}^{1}$, ${\text{A}}_{\text{g}}^{7}$, and ${\text{A}}_{\text{g}}^{12}$, exhibited distinct thickness dependence of the in-plane Raman anisotropy, whereas Raman anisotropy of the rest of the A_g_ modes and the B_g_ modes is insensitive to the flake thickness. The observed anisotropies were comprehensively understood by invoking the interference effect and the semiclassical complex Raman tensor analysis.

## Method

### Synthesis of GaTe crystal

High crystallinity GaTe single crystals were synthesized by melting the stoichiometric mixture of high-purity raw elements (99.99%, Alfa Aesar) using a temperature gradient technique, as specifically described elsewhere^40,41^. Firstly, the constituent elements were weighed in the desired molar ratio, then loaded into quartz ampoule, and evacuated under the pressure of 10^–3^ Torr. The ampoule was placed in a vertical furnace, then heated up to 1223 K following the three-step temperature profile from 300 to 773 K, 773 to 1023, and 1023 to 1223 K with the heating rates of 50, 10, and 5 K h^−1^, respectively, to prevent the explosion risks due to high vapor pressure of tellurium. After soaking this temperature for 33 h, the ampoule was slowly cooled at a precise rate of 1 K h^−1^ to 1023 K and finally followed by a cool down to room temperature at a rate of 30 K h^−1^.

### Sample preparation

Thick GaTe flakes were mechanically exfoliated from the bulk single-crystal samples and then transferred onto 275 nm SiO_2_/Si substrates. The exfoliated flakes were immediately placed in the closed-cycle cryostat with the pressure of 10^–3^ Torr in order to prevent oxygen contamination.

### Raman characterization

Raman and photoluminescence (PL) spectra were measured in the backscattering geometry by using 532 nm laser line as an excitation light source. The polarization configuration was either parallel polarization configuration or perpendicular polarization configuration. Polarization anisotropy was investigated by rotating the polarization directions of both the incident and the scattered light with respect to the crystal y-axis. The linearly polarized Raman spectra were measured by using an Acton Spectra Pro 2500i system with the 532 nm laser line, a 50× objective, and 1200 lines/mm grating. The laser spot size was ~ 1 μm and the laser power was 400 μW.

## Supplementary Information

Supplementary Information.
