# Supplementary material for: Thickness-dependent in-plane anisotropy of GaTe phonons
Source: Sci Rep. 2021 Oct 27;11:21202. doi: 10.1038/s41598-021-00673-0 (PMC8551200; doi:10.1038/s41598-021-00673-0)
Supplement: Supplementary file 1 — Supplementary Information. [file 41598_2021_673_MOESM1_ESM.pdf]

Supplementary Information

Thickness-dependent in-plane anisotropy of GaTe phonons

Nguyen The Hoang[1], Je-Ho Lee[1], Thi Hoa Vu[2], Sunglae Cho[2*], and Maeng-Je Seong[1,3*]

[1] Department of Physics, Chung-Ang University, Seoul, 06974, Republic of Korea

[2] Department of Physics and Energy Harvest Storage Research Center, University of Ulsan, Ulsan, 44160, Republic of Korea

[3] Center for Berry Curvature-based New Phenomena, Chung-Ang University, Seoul, 06974, Republic of Korea

[*] To whom correspondence should be addressed: slcho@ulsan.ac.kr and mseong@cau.ac.kr

Table **S1**. Calculated and experimentally observed vibrational frequencies of all the phonon modes in Gallium Telluride (GaTe). Raman-inactive vibrational modes and their frequencies are shown in black.

	Calc. (cm^{-1}) Huang[1].	Symmetry	Exp. (cm^{-1})			Calc. (cm^{-1}) Huang[1].	Symmetry	Exp. (cm^{-1})
1	0	B_u			19	142.4	B_u	
2	0	A_u			20	155.2	A_g^7	152.5
3	0	B_u			21	163.2	B_u	
4	40.4	A_g^1	40.2		22	167.6	B_g^4	163.0
5	41.8	B_u			23	168.6	A_u	
6	42.2	B_g^1			24	172.7	B_g^5	
7	51.6	A_u			25	173.6	A_u	
8	51.7	A_g^2	51.5		26	178.2	A_u	
9	58.2	B_g^2	56.5		27	178.4	B_g^6	
10	64.7	B_g^3			28	181.7	A_g^8	176.9
11	66.8	A_g^3	66.2		29	198.1	B_u	
12	69.7	A_u			30	206.6	B_u	
13	75.5	A_g^4	75.6		31	211.4	A_g^9	208.9
14	90.3	B_u	90.3		32	212.8	A_g^{10}	208.9
15	91.9	B_u			33	216.3	B_u	
16	109.0	A_g^5	109.5		34	271.0	A_g^{11}	269.0
17	114.4	A_g^6	115.0		35	280.1	B_u	
18	116.0	B_u			36	289.2	A_g^{12}	283.8

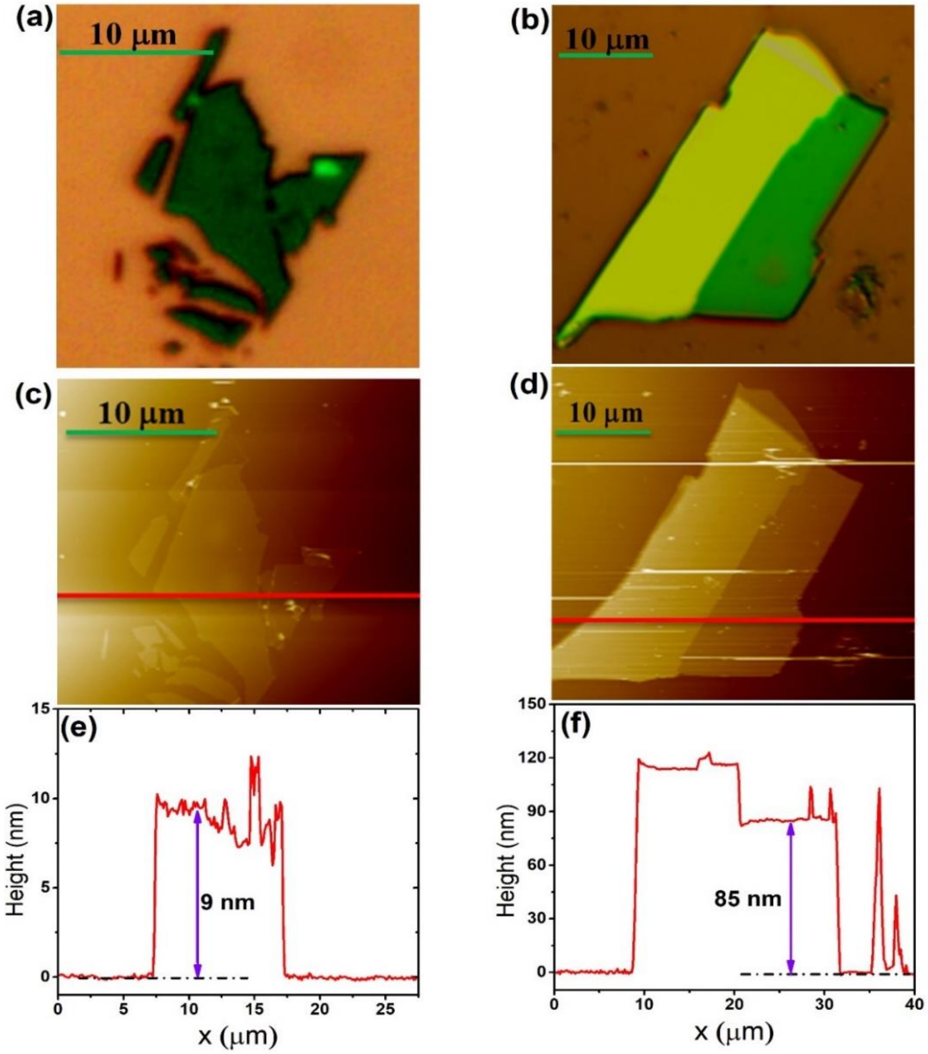

Figure **S1.** (a), (b) Optical microscope images (c), (d) AFM images, and (e), (f) AFM height profiles of 9 nm and 85 nm thick GaTe flakes, respectively.

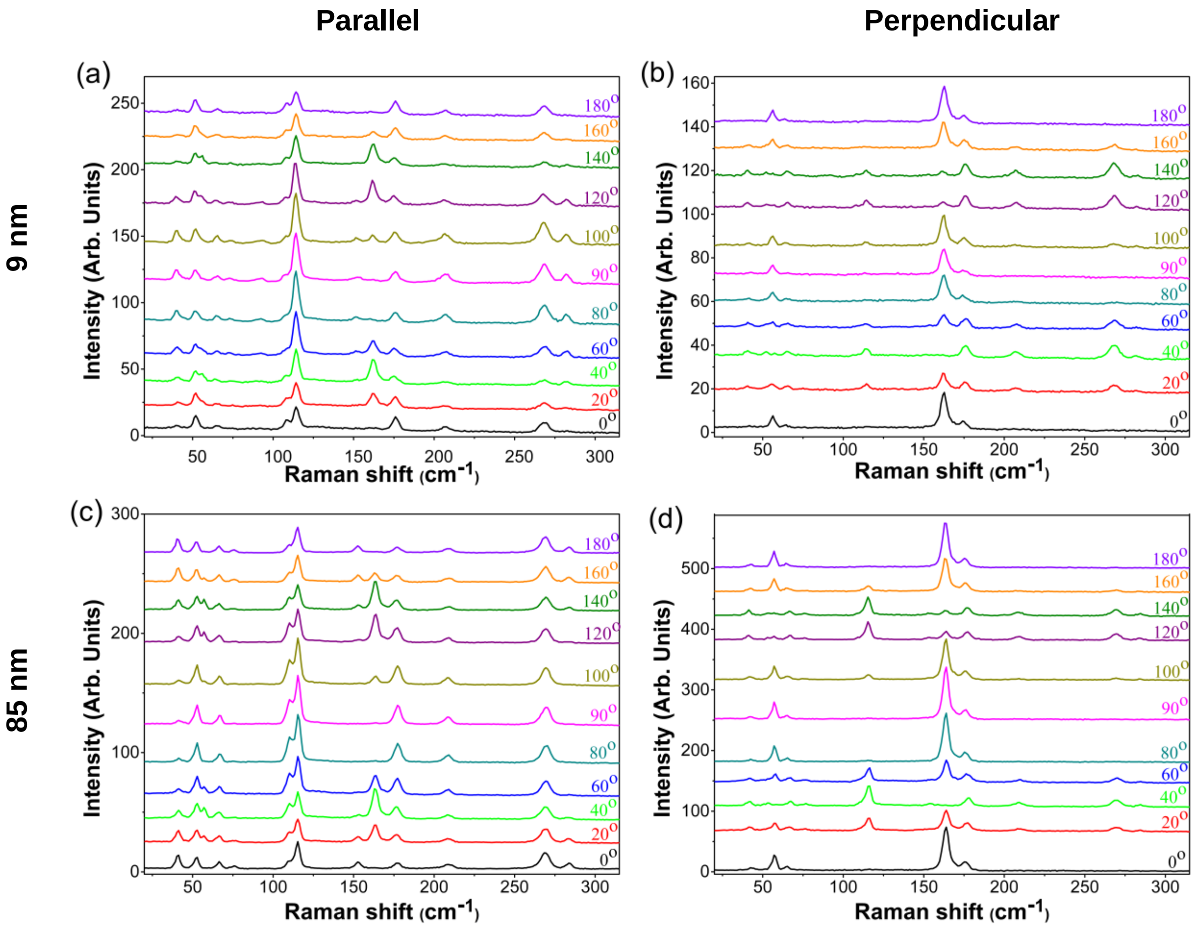

Figure **S2**. Angle-resolved polarized Raman spectra of two flakes with different thicknesses of 9 nm and 85 nm, respectively, under parallel and perpendicular polarization configurations.

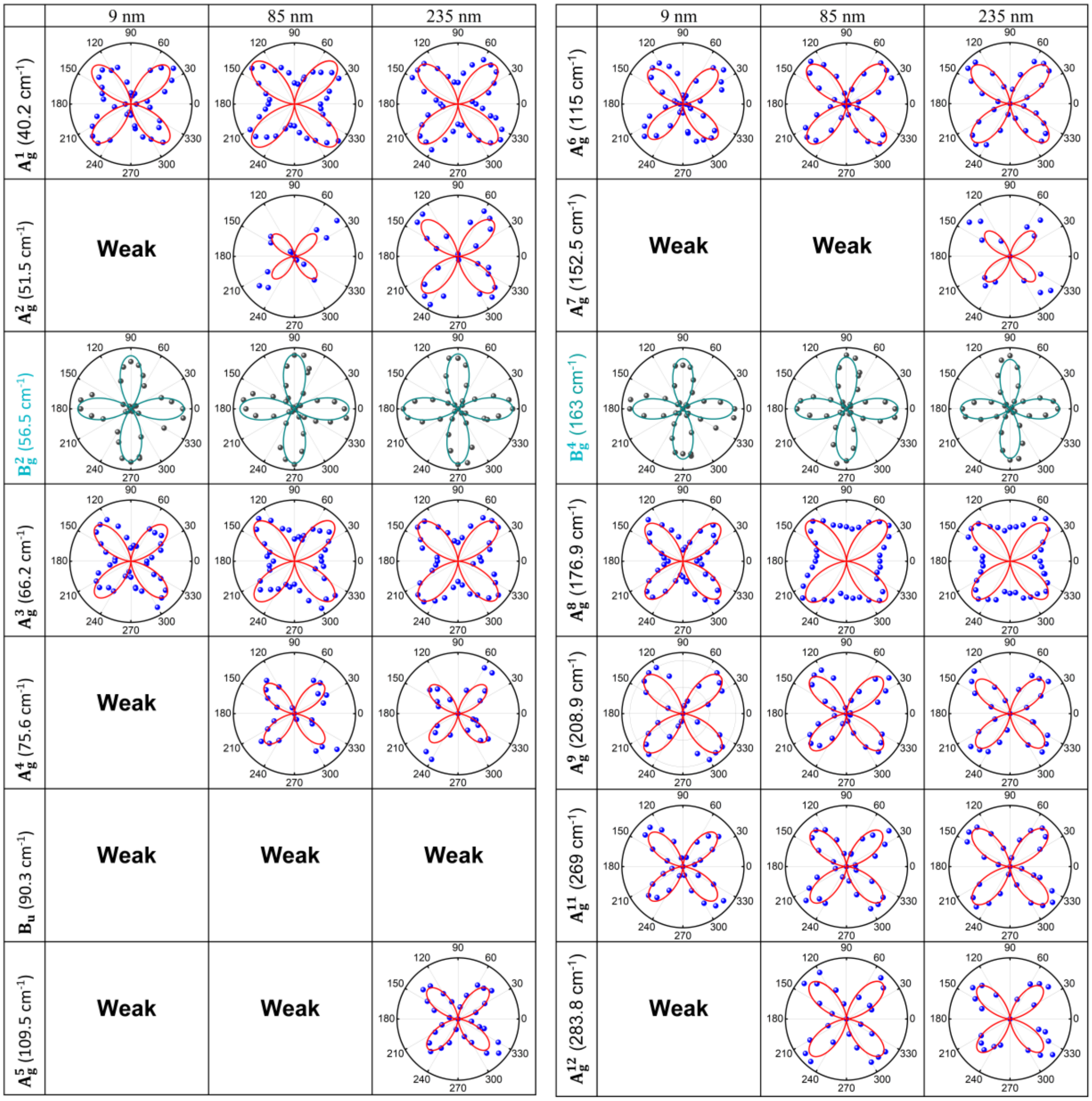

Figure **S3.** Raman intensity polar plots of three flakes with different thicknesses of 9 nm, 85 nm, and 235 nm, with 532 nm laser excitation under perpendicular polarization configuration. The solid red and cyan curves are fitted lines, and the dots are experimental values.

$$I\,(A_g^{\perp}) \sim |c|^2\cos^2\theta\,\sin^2\theta + |b|^2\sin^2\theta\,\cos^2\theta - 2|b||c|\cos^2\theta\,\sin^2\theta\,\cos\psi_{bc}$$

$$\rightarrow I\,(A_g^{\perp}) \sim \frac{1}{4}\left(|c|^2 + |b|^2 - 2|b||c|\,\cos\psi_{bc}\right)\sin^2 2\theta \qquad (2\sin\theta\,\cos\theta = \sin 2\theta)$$

$$I\,(B_g^{\perp}) \sim (|f|^2\cos^2 2\theta)$$

We can rewrite the equation above:

$$I\,(A_g^{\perp}) \sim \sin^2 2\theta \qquad (1) \qquad\qquad I\,(B_g^{\perp}) \sim \cos^2 2\theta \qquad (2)$$

1. Raman intensity

$$I_\nu(E_L) = \left|\sum_{i,m,m'} \frac{\langle f|H_{op}|m'\rangle\langle m'|H_{ep}^{\nu}|m\rangle\langle m|H_{op}|i\rangle}{(E_L-\Delta E_{mi})(E_L-\hbar\omega_\nu-\Delta E_{m'i})}\right|^2 \quad (1)$$

where i and f represent initial and final states; E_L is the laser photon energy; ΔE_{mi}=E_m-E_i-$i\gamma$, m and m$'$ are intermediate states; γ is the broadening factor. $\langle m|H_{op}|i\rangle$ and $\langle f|H_{op}|m'\rangle$ correspond to optical absorption and emission processes, which related to the electron-photon interaction, whereas $\langle m'|H_{ep}^{\nu}|m\rangle$ represents the electron-phonon interaction.

2. Interference effect.

As black phosphorus (BP)[2], the interference effect also contributes strongly to Raman scattering anisotropy. This effect depends on the polarization of light due to the different refractive indices in the main axes (y-axis and z-axis) of GaTe. The net enhancement of incident light F_{ab} caused by multiple reflections at a position x measured from GaTe surface is given by[2–4]:

$$\boldsymbol{F_{ab}(x)} = t_{01}\frac{\left(1+r_{12}r_{23}e^{-2i\beta_2^{ex}}\right)e^{-i\beta_2^{ex}}+\left(1+r_{12}r_{23}e^{-2i\beta_2^{ex}}\right)e^{-i(2\beta_1^{ex}-\beta_x^{ex})}}{1+r_{12}r_{23}e^{-2i\beta_2^{ex}}+\left(1+r_{12}r_{23}e^{-2i\beta_2^{ex}}\right)r_{01}e^{-2i\beta_1^{ex}}} \quad (2)$$

Where $t_{ij} = 2\,n_i/(n_i+n_j)$ and $n_{ij} = (n_i-n_i)/(n_i+n_j)$ are Fresnel transmittance and reflectance coefficients, respectively. The interfaces of the *i*th and *j*th layer with *i, j* indices given by air (0), GaTe (1), SiO_2 (2), and Si (3) they describe in Figure S5. n_i is the complex refractive index of the ith layer. β_x^{ex}=$2\pi x n_i/\lambda_{ex}$ and β_i^{ex}=$2\pi d_i n_i/\lambda_{ex}$ are phase factors with d_i the thickness of the ith layer and λ_{ex} the excitation wavelength.

The net enhancement of scattered light $F_{sc}(x)$ due to multiple reflections at a position x measured from the GaTe surface is calculated by[2–4]:

$$\boldsymbol{F_{sc}(x)} = t_{01}\frac{\left(1+r_{12}r_{23}e^{-2i\beta_2^{sc}}\right)e^{-i\beta_2^{sc}}+\left(1+r_{12}r_{23}e^{-2i\beta_2^{sc}}\right)e^{-i(2\beta_1^{sc}-\beta_x^{sc})}}{1+r_{12}r_{23}e^{-2i\beta_2^{sc}}+\left(1+r_{12}r_{23}e^{-2i\beta_2^{sc}}\right)r_{01}e^{-2i\beta_1^{sc}}} \quad (3)$$

Where β_x^{sc}=$2\pi x\boldsymbol{n_i}/\lambda_{sc}$ and β_i^{sc}=$2\pi\boldsymbol{d_i n_i}/\lambda_{sc}$ are the phase factors for the scattered light with a wavelength λ_{sc} related to the Raman shift of a particular spectrum. The total enhancement factors are then given by:

$$F = N\int_0^{d1}|F_{ab}(x)\,F_{sc}(x)|^2\,dx \quad (11)$$

Where N is the normalization constant. The measured Raman intensity is $I = I_i \times F$, where I_i is the intrinsic Raman intensity, including only electron-photon and electron-phonon interactions.

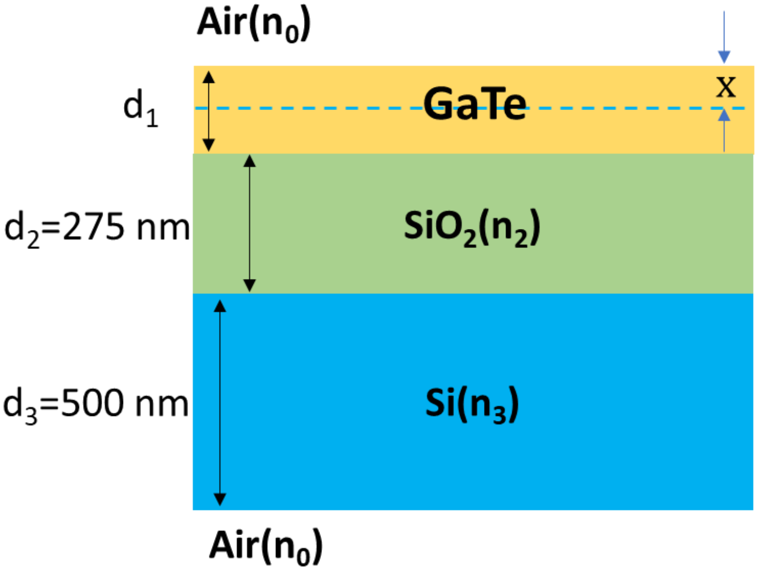

Figure **S4.** Schematic diagram of the sample structure used for absorption spectra and enhancement factor calculations.

Table **S2.** Laser excitation wavelength of the complex refractive index $\tilde{n}$ of bulk GaTe for y- and z-axis, SiO_2[1], and Si [1]

GaTe-x	GaTe-y	SiO_2	Si
3.70-0.515i	3.87-0.211i	1.46	4.21-0.010i

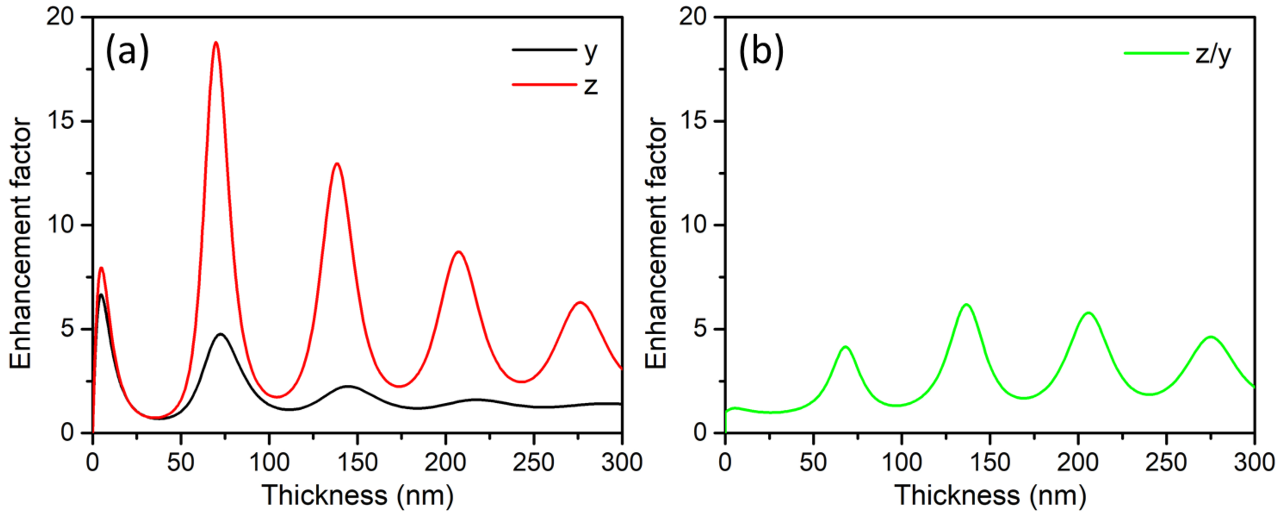

Figure **S5.** Calculated results for enhancement factor. (a) enhancement factor along y- and z-axis. (b) the ratio of enhancement factor along z-axis over that along y-axis.

References

1. Saito, R. *et al.* In-Plane Optical Anisotropy of Layered Gallium Telluride. *ACS Nano* **10**, 8964–8972 (2016).

2. Ling, X. *et al.* Anisotropic Electron-Photon and Electron-Phonon Interactions in Black Phosphorus. *Nano Lett.* **16**, 2260–2267 (2016).

3. Zhang, H. *et al.* Interference effect on optical signals of monolayer MoS_2. *Appl. Phys. Lett.* **107**, 8–12 (2015).

4. Yoon, D. *et al.* Interference effect on Raman spectrum of graphene on SiO_2 /Si. *Phys. Rev. B - Condens. Matter Mater. Phys.* **80**, 1–21 (2009).
